# Periodic Fever in Children: Etiology and Diagnostic Challenges

**DOI:** 10.7759/cureus.27239

**Published:** 2022-07-25

**Authors:** Paola Carolina Espin Diaz, Kawaljeet Singh, Pawani Kher, Chaithanya Avanthika, Sharan Jhaveri, Yosra Saad, Shankhaneel Ghosh

**Affiliations:** 1 General Practice, Universidad Internacional del Ecuador, Quito, ECU; 2 Medicine and Surgery, Guru Gobind Singh Medical College and Hospital, Faridkot, IND; 3 Medicine and Surgery, Punjab Institute of Medical Sciences, Jalandhar, IND; 4 Medicine and Surgery, Karnataka Institute of Medical Sciences, Hubballi, IND; 5 Internal Medicine, Smt. Nathiba Hargovandas Lakhmichand Municipal Medical College, Ahmedabad, IND; 6 Medicine and Surgery, University of Medical Sciences and Technology (UMST), Khartoum, SDN; 7 Medicine and Surgery, Institute of Medical Sciences and SUM Hospital, Bhubaneswar, IND

**Keywords:** pfapa, non-hereditary periodic fever, hereditary periodic fever, children, periodic fever

## Abstract

Periodic fever in children is an autoinflammatory illness with an unknown cause. Symptoms include frequent episodes of fever that are followed by an increase in inflammatory markers. A genetic background for periodic fever of unknown origin has been hypothesized, based on its family clustering and parallels to other autoinflammatory illnesses such as familial Mediterranean fever. Genome analysis has been used in studies to look for related gene variations in periodic fever of unknown origin in the pediatric population.

Children with periodic fevers might be a diagnostic challenge. After ruling out the most prevalent causes, a wide variety of other possibilities are investigated. Infectious and noninfectious causes of periodic fever in children are discussed in this article.

Inflammasomes (intracellular proteins that activate interleukin (IL)-1b and IL-18) and genetic/hereditary variations are thought to be implicated in the pathogenesis of periodic fever. Evaluation and ruling out possible infective or noninfective causes is vital in the diagnosis of periodic fever in children. Investigations demonstrate that there isn't a single gene linked to it, suggesting that it may have a multifactorial or polygenic origin, with an environmental trigger causing inflammasome activation and fever flares. Treatment is usually symptomatic, with drugs such as colchicine and cimetidine having shown promising results in trials.

We explored the literature on periodic fever in children for its epidemiology, pathophysiology, the role of various genes and how they influence the disease and associated complications, and its various treatment modalities.

## Introduction and background

Periodic fever syndrome is a group of autoinflammatory disorders presenting as recurrent episodes of fever accompanied by inflammation of the eyes, joints, skin, or serosal surfaces [1]. It is a pediatric syndrome that affects both sexes, with slightly more male predominance, and has no predilection for any particular ethnic or racial group [1-3]. Considering the molecular mechanisms, these autoinflammatory diseases can be categorized into Inflammasomopathies or IL-1 β-activation syndromes including familial Mediterranean fever (FMF), cryopyrin-associated periodic syndromes (CAPS), and mevalonate kinase deficiency (MKD), and protein folding disorders such as tumor necrosis factor (TNF) and receptor-associated periodic fever syndrome (TRAPS) [4,5,6].

 In 1987, Marshall et al. discovered an unknown periodic fever syndrome, which was later termed periodic fever, aphthous stomatitis, pharyngitis, cervical adenitis (PFAPA) syndrome. It is a recurrent fever syndrome that presents at a young age, especially in children less than five years of age [7,8]. Although the pathogenesis is not clear, it is believed that tonsils are the primary immune dysregulation point [9]. In 1997, Mediterranean Fever (*MEFV*) was the first gene discovered for causing familial Mediterranean fever, the most common hereditary cause of periodic fever [1]. Since there is no specific genetic test or confirmatory laboratory test, diagnosis is largely based on clinical features. 

The treatment of periodic fever relies on the suppression of systemic inflammation through the administration of corticosteroids. Interleukin (IL) 1 has a predominant role in innate immunity and inflammasome formation; it is also one of the important target treatments [10]. 

In this review, we provide a more detailed look at disease-specific pathophysiology, epidemiology, diagnostic criteria, differential diagnosis, and treatment options for periodic fever in children.

## Review

Pathophysiology and epidemiology of periodic fever syndrome

PFAPA syndrome, initially delineated in 1987 by Marshall et al., is one of the most common periodic fever syndromes in children. PFAPA is not an inheritable malady and the clinical presentation was recently mentioned in a 301 patients cohort study by Renko et al. The disease presents at a median age of four years with perennial fever, exudative redness with negative throat culture, cervical lymphadenopathy, and oral aphthae. It less frequently presents with abdominal pain, arthralgia, myalgia, and headache [11-13]. Regardless of antibiotics or antipyretics, each step lasts four to six days and is repeated every three to five weeks. Approximately 30% of PFAPA patients are predominantly male (M/F=1.8: 1) with a positive family history. There are no specific laboratory tests to diagnose PFAPA. However, leukocytosis and high C-reactive protein (CRP) levels predominate during a flare, with normalization between episodes [11-14]. Previous studies have shown that *MEFV* gene variants may have a modifier role in PFAPA. PFAPA is taken into account as self-limiting since the disease usually tends to change state and goes away once the patient is about 12 years of age [11-14].

The incidence of PFAPA, as mentioned by a study in Norway, is 2.3/10,000 each year in children up to five years of age. This makes PFAPA one of the most common pediatric periodic fever syndromes. PFAPA occurs in both sexes, with a slightly higher male predominance of 55-65% [2,14-16]. The syndrome has been reported in patients of various ethnicities [3,15-18].

Familial Mediterranean fever is one of the most prevalent inheritable inflammatory diseases. The main sequence in patients with familial Mediterranean fever is the *MEFV* gene, a secret writing pyrin macromolecule. Pyrin is a physiological protein within the nucleotide-binding oligomerization domain, leucine-rich repeat, and pyrin domain-containing three (NLRP3) inflammasome complex; mutation of this protein causes excess inflammation via caspase-1 activation and IL-1b production [11]. A number of mutations are related to dominant inheritance. The most common problematic variants are all settled in desoxyribonucleic acid 10: *M694V, M694I, M680I, V726A, R761H,* and *A744S*. M694V is the most typical damaging variant and is related to a severe expression of the disease [2,19]. Familial Mediterranean fever is recognized worldwide; however, it is far more common among populations originating from the eastern Mediterranean region [2,20].

CAPS pathophysiology is related to *NLRP3* mutation, coding cryopyrin. *NLRP3* may be a key supermolecule of the *NLRP3* inflammasome that is activated by many stimuli and causes the conversion of pro-IL-1 to mature IL-1 by caspase-1 activation. It is located on chromosome 1q44 and encodes for cryopyrin. Recently, *NLRP3*-associated autoinflammatory disease was suggested as a new name for CAPS. CAPS is a heritable dominant disorder. The common clinical options are fever, urticarial rash, system symptoms, elevated acute part reactants, and conjunctivitis [11-10]. CAPS is a rare disease, which affects one to three per million with no gender or ethnic predisposition [2,21-23]. 

TRAPS was initially rumored in a very massive Irish/Scottish family and was originally delineated as familial Hibernian fever in 1982. When the molecular basis of the disease showed association with mutations within the cistron of TNF receptor taxon member 1A (*TNFRSF1A*), it was renamed TRAPS [11,24-26]. The two most common *TNFRSF1A* variants, *P46L* and *R92Q*, are present in approximately 10% of West Africans and 2% of Caucasians, respectively. The majority of carriers of those two are variants unaffected, and the way they cause disease in a minority remains obscure [27]. TRAPS has a calculable prevalence of roughly one to two per million. It has been additionally often said to be more prevalent in Caucasians; however, this actually reflects ascertainment bias [2,27-29].

Cyclic neutropenia is an uncommon hematologic condition marked by recurrent episodes of fever, mouth ulcers, and infections [30]. The oscillatory generation of cells by the bone marrow is responsible for blood cell fluctuations [30]. Autosomal-dominant neutropenia and sporadic occurrences of the disease are caused by a mutation in the neutrophil elastase (*ELA2*) gene, which is located at 19p13.3, according to genetic, molecular, and cellular investigations [31].

Hyper-IgD syndrome, also known as mevalonate kinase-associated periodic fever syndrome, is a genetic disease with autosomal recessive inheritance. It is caused due to a missense mutation in the mevalonic kinase (*MVK*) gene. Most cases are found in Western Europe and present within the first year of life [31-32]. 

Clinical features and associated clinical syndromes

Periodic fever can be defined as repetitive episodes of fever with no symptoms in between the episodes. These episodes generally last for a few days to weeks up to several months [32,33]. These disorders present with fever as the primary symptom and an associated, similar set of accompanying symptoms. The episodes may be associated with "clockwork" periodicity or may be irregular. Many periodic fever syndromes are a result of a genetic mutation resulting in a defective enzyme or protein that leads to unique clinical manifestations and, hence, has hereditary meaning [34]. Some also have an ethnic or geographic dominance [35].

Periodic fever syndromes can be broadly classified into two categories:

1. Hereditary, including familial Mediterranean fever, TRAPS, Hyper-IgD syndrome, CAPS, chronic infantile neurological, cutaneous, articular syndrome(CINCA), Muckle-Wells syndrome, Blau syndrome, and familial cold autoinflammatory syndromes (FCAS) [36-38].

2. Non-Hereditary, including PFAPA syndrome, cyclic neutropenia, and Castleman's disease [38-40].

Familial Mediterranean Fever

It is the most common autoinflammatory disease prevalent amongst people of Mediterranean and Middle Eastern descent. An astonishing feature of familial Mediterranean fever is that despite repeated severe multisystem attacks, it often does not result in long-term drastic complications [3]. Symptoms of familial Mediterranean fever begin between 2-10 years of age with 20% affected before two years of age and two-thirds affected before 10 years of age [34]. The main symptoms of the disease are fever, abdominal pain, and arthritis. The temperature rises to about 40 degrees celsius, associated with chills, and lasts from 12 hours to three days. Abdominal pain may occur suddenly before the fever, mimics appendicitis, and could be accompanied by diarrhea. Asymmetrical monoarthritis, which compromises the ankle, knee, or wrist joint, usually resolves in 5-14 days. An erysipelas-like rash may be present over the affected joint-in about 25% affected, leading to the misdiagnosis of juvenile idiopathic arthritis. The rash is unilateral and disappears within two to three days. Pleuritic chest pain (may be associated with difficulty in breathing), peritoneal abdominal pain, and pericarditis (in <1%) can also be present. Splenomegaly may be present in 30-50% of the affected. Amyloidosis is the most common complication and cause of mortality of untreated familial Mediterranean fever and can progress to end-stage renal disease [3,15,34,37].

TRAPS

Initially referred to as familial Hibernian fever, TRAPS is a very rare disease affecting almost one or two in a million, with a predilection for Caucasians and Asians [3]. It demonstrates an autosomal dominant mode of inheritance with the median age of presentation around seven years. The patient presents primarily with fever and painful erythema. The fever recurs every four to six weeks and lasts anywhere from five days to three weeks. Episodes are precipitated by infections, emotional and physical stress, trauma, and hormonal changes. Fever is associated with chills and intense muscle pain (myalgias). A migratory painful erythema/rash that moves centrifugally, from arms and legs to the body, is usually present. A skin biopsy may reveal lymphocytic infiltrates [38-41]. Ophthalmologic manifestations include conjunctivitis, periorbital edema, and periorbital pain may also appear. Less specific but common symptoms include testicular pain, splenomegaly, abdominal pain with nausea and vomiting, and serositis (peritonitis, pleuritis, and pericarditis). Like familial Mediterranean fever, untreated TRAPS can lead to amyloid deposition, which increases mortality and morbidity by causing proteinuria and kidney failure [3,15,34,37,39].

Hyperimmunoglobulin D syndrome (HIDS)

HIDS is variable in severity and occurs with irregular periodicity. The severity and frequency of attacks decrease with increasing age [11]. Mevalonate aciduria is the severe form of HIDS at birth and presents with neurological conditions, mental retardation, failure to thrive, and a high rate of stillbirth [11]. Symptoms of the less severe variants of HIDS are episodic fever associated with skin rash and chills and painful ulcers in the mouth or vagina. The fever is sudden in onset and lasts for four to six days. Fever may be provoked by stress, vaccination, or infections. The infant may also be irritable or aggressive. Prominent cervical lymphadenopathy is commonly palpated but swelling of lymph nodes may also be present in other parts of the body. Arthralgias and petechial rash on extremities are also frequent findings. Headache, abdominal pain, nausea, diarrhea, and serositis may also be associated. Rare features include macrophage activation syndrome, retinitis pigmentosa, and amyloidosis [3,15,34,37,41].

CAPS

CAPS is an autosomal dominant disease with no gender or ethnic predisposition. It is a rare disease and affects one to three infants per million. The symptoms present since birth and occur daily, which get worsened in a diurnal manner. The main symptoms of CAPS are fever associated with fatigue, conjunctivitis, and urticarial rash. The rash is non-pruritic and increases in intensity with fever. Irreversible symptoms include sensorineural hearing loss, vision loss, skeletal deformities, cognitive impairment, and systemic amyloidosis [3,15,34,41,42-4 3].

Cyclic Neutropenia

Cyclic neutropenia is a rare, autosomal dominant hematologic disorder that presents as a consequence of a periodic decrease in the absolute neutrophil count (<200 cells/cumm). It is present in infants(<1 year old) and has clockwork periodicity, meaning that the symptoms recur after every 21 days. Cyclic neutropenia presents as recurrent fever due to superimposed bacterial infections as a result of decreased neutrophil count. Deep and painful mouth ulcers last for one week and are also associated with pharyngitis. Lymphadenopathy, respiratory, skin infections, gingivitis, periodontitis, bacterial otitis media, and sinusitis are common. Life-threatening bacterial complications such as spontaneous peritonitis, segmental bowel resection, and septicemia can also be seen especially during episodes of neutropenia [34,44-47].

PFAPA Syndrome: 

PFAPA syndrome presents in children <5 years old with a predisposition to the male gender. Symptomatic bouts last for between three to seven days and demonstrate clockwork periodicity, i.e., recur every two to eight weeks. Interestingly, patients with PFAPA present with a decent general condition despite running a high fever. The clinical presentation of PFAPA is associated with the abrupt onset of fever, which suddenly resolves in three to four days. Fever can be as high as 39-40 degrees celsius, and is immune to treatment with antipyretics or antibiotics, but is responsive to steroids. Aphthous stomatitis, which is usually complicated by oral candidiasis and infective gingivostomatitis, may also be present. Tonsillitis, with a negative strep culture, is characteristic. Lymphadenopathy, headache, general malaise, and bone pain are also commonly associated symptoms. Less commonly, abdominal pain, aphthae, and arthralgias might also be present [3,15,38,48-50].

Diagnostic protocol and differential diagnosis

Diagnostic Protocol

The diagnosis of periodic fever has always been a challenge. In 1989, Thomas et al. proposed the first diagnostic criteria for PFAPA [15]. Over the past few decades, more studies and evaluations were performed to come up with better diagnostic criteria. In 1999, a modified Marshall`s criteria were set [15] but it had some drawbacks. Vanoni et al. had defined more symptoms in their criteria [51-52], which had been used together with Marshall`s criteria. The use of these criteria depended on the physician's preference [53]. The gold standard diagnosis criteria are shown in Table 1 [15].

**Table 1:** Standard for diagnosis of PFAPA syndrome PFAPA: periodic fever, aphthous stomatitis, pharyngitis, cervical adenitis

Age of presentation is <5 years
Associated symptoms include aphthous stomatitis, adenitis, or pharyngitis with the absence of upper respiratory tract infection
Other periodic fever syndromes are excluded e.g cyclic neutropenia
Normal growth and development
No symptoms in between fever attacks

In addition to the above criteria, other indicators were used to help in the diagnosis. These were having full responses to one to two doses of corticosteroids during the treatment and other family members being well during fever episodes of the patient. A study done by Manthiram et al. showed that 80% of physicians acknowledged that to diagnose PFAPA, the patients would have a periodic fever, be symptom-free in between, and have normal growth and development [54], while 17% expected all of Marshall`s modified criteria to be shown to diagnose PFAPA.

The involvement of clinical experts including immunologists and pediatric infectious specialists would help with the diagnosis [55]. Despite strong evidence showing PFAPA as an immune-mediated disease, some studies suggested the possibility of a genetic element [56-57]. The workup for these patients will start with a thorough history of the fever, duration, symptom-free intervals, presence of any associated symptoms, and medication effect on these episodes. It would also include family history and looking for any genetic causes for periodic fever. This would be followed by a full examination of the patients looking for lymph nodes, mouth ulcers, skin rash, or signs of joint inflammation, which may suggest autoimmune disease. Other findings like faltering growth would suggest a different diagnosis. Laboratory investigations play a role in the diagnosis. These would include complete blood count (CBC), CRP, Erythrocyte sedimentation rate (ESR), and ferritin. Raised inflammatory markers would support the diagnosis of recurrent viral infections instead of PFAPA [53]. Special investigations like immunoglobulins and vaccine antibody titers would be useful to exclude immunodeficiencies [55].

Differential Diagnosis

PFAPA is the most common cause of periodic fever; however, it does not have a diagnostic test. The majority of the other syndromes have a confirmatory genetic test except for cyclic neutropenia which is diagnosed by low neutrophil count. Multiple reports on hereditary periodic fever were written [35,58]. Furthermore, the consideration of hereditary fever syndromes depends on the progenitors of the patients [37,59]. 

The Eurofever study done in 2015, has proposed new classification criteria for monogenic inflammatory diseases (AIDs). PFAPA patients were chosen as the control group in this study. The results were then used in addition to the Gaslini score (employed to assess the probability of carrying mutations for monogenetic fevers) to identify patients with suspected inflammatory disease depending on clinical manifestations [60].

Butbul-Aviel et al. performed a study in Israel where 70% of the patients had familial Mediterranean fever genes due to their Mediterranean ancestry. Out of the 270 PFAPA patients enrolled in the study, 51 were also found to have familial Mediterranean fever [61].

The disorders described below are the ones with the fundamental features of periodic fever and are shown in order of frequency in Table 2 [34].

**Table 2:** Frequency order of disorders presenting fundamental features of periodic fever HIDS: hyperImmunoglobulinemia D; MVK:  mevalonate kinase deficiency; TRAPS: tumor necrosis factor receptor-associated syndrome; CAPS: cryopyrin-associated periodic syndromes; PFAPA: periodic fever, aphthous stomatitis, pharyngitis, cervical adenitis

Syndrome	Cause
PFAPA syndrome	Unknown
Cyclic neutropenia	Enzyme defect
Familial Mediterranean fever	Protein defect
HIDS/MVK	Enzyme defect
TRAPS	Protein defect
CAPS	Protein defect

To differentiate between these disorders, laboratory investigations and genetic testing are performed. PFAPA patients have elevated ESR but <60mm/hr, as well as mild neutrophilia. Elevation of ESR is seen only while the febrile episode lasts [38]. Elevated acute phase reactants are commonly seen in familial Mediterranean fever. A missense mutation in the *MEFV* gene in chromosome 16 results in decreased production of pyrin [62-64]. HyperIgD syndrome, also known as MKD, is an autoinflammatory disorder. HIDS and MVK are linked together because initially IgD was raised during the febrile episodes. However, this was not the case in 20% of the patients [65-66]. There is a variable increase in IgD and IgA, elevated acute phase reactants as well as increased urinary mevalonic acid [67]. A variable decrease in serum cholesterol is detected in those patients. The gene mutation is known as *MVK* mutation, which is found in chromosome 12. This leads to decreased mevalonate kinase activity and isoprenoids. In cyclic neutropenia, blood tests would result in an absolute neutrophil count of < 500 cells / mm3 for three to five days. Upon genetic testing, the ELA2 mutation is found in chromosome 19. This leads to mutant neutrophil elastase [66]. In TRAPS, elevated acute phase reactants are shown in blood tests. The gene responsible for this defect is found in chromosome 12. It leads to a mutation in *TNFRSF1A*, which reduces soluble TNF receptor superfamily type 1A [32]. As researchers developed new studies to detect mutations associated with CAPS, newer somatic mutations have been discovered. One study reported that 19% of CAPS-like patients had shown evidence of mosaicism [68,69]. Thus, the importance of genetic testing is highlighted [70]. Clinical diagnosis would be helpful in cases where there is mosaicism. This would be shown as elevated acute phase reactants along with at least two clinical features: cold-triggered episodes, sensorineural hearing loss, chronic aseptic meningitis, urticaria-like rash, and skeletal abnormalities. By using this criteria, sensitivity and specificity were found as 81% and 94%, respectively [36]

Treatment

PFAPA Treatment

Since this periodic fever syndrome is a benign condition, treatment is usually optional. The following interrogants should be considered by caregivers if they choose not to initiate medical treatment [71]: (i) Can the child afford to miss several days a month of school? (ii) Can the caregiver miss work to care for a child with fever? (iii) Does familial distress related to the illness favor the potential complications of therapy? The main goal of the treatment of PFAPA is to significantly improve the child's quality of life until spontaneous resolution happens [72-75].

**Table 3:** Proposed treatments for PFAPA NSAIDS: non-steroidal anti-inflammatory drugs; PFAPA: periodic fever, aphthous stomatitis, pharyngitis, cervical adenitis

Medication	Indication	Observation
Glucocorticoids (Prednisone, Betamethasone)	Interruption of recurrences	Very good response (Level of evidence 2b)
Colchicine	Prophylactic treatment	Gastrointestinal side effects (Level of evidence 1b)
Cimetidine	Prophylactic treatment	Efficacy not proven
Paracetamol	Symptomatic relief	Low efficacy
NSAIDS	Symptomatic relief	Good efficacy for fever
Anakinra (IL-1 antagonist)	Interruption of recurrences	Tested on few patients (4 case series)

Glucocorticoids 

The most commonly used first-line treatment is low-dose corticosteroids [2,72,75-76]. The dose currently used is 1-2 mg/kg orally given at fever onset. A second dose can be administered if the fever recurs within 48 hours and if only 1 mg/kg was given. The percentage of patients that require a second dose of corticosteroids is 20-25%. Aside from the adverse effects of corticosteroids, it has been documented that this treatment can cause a shortening of the interval between attacks, in 19 to 50% of patients [71].

Prophylactic Therapy with Colchicine and Cimetidine

This approach is usually recommended in patients that, despite the use of glucocorticoids, continue to have febrile episodes. The administration of the following doses of colchicine: 0.5 mg/day if < 5 years of age, 1 mg/day if 5-10 years, 1.5 mg/day if > 10 years, showed a decrease in febrile attacks. This effect was found to be significant in patients with MEFV variant [72-73]. The effectiveness of cimetidine (20 mg/kg or 300 mg/day) in decreasing flares has been reported by low evidence studies like case reports, case series, and small cohort studies [71, 73].

Tonsillectomy

A surgical approach to PFAPA is recommended in children in whom despite medical treatment, illness has a considerable impact on quality of life [74-75]. The effectiveness in reducing the number of flares in patients that underwent tonsillectomy with or without adenoidectomy for PFAPA has been documented since 1999 with some small sample randomized controlled trials, and several case series [72]. In a literature review performed by Førsvoll et al. in 2016, a positive result was found in patients after tonsillectomy and adenotonsillectomy in 523 out of 555 children [75]. Karayağmurlu's study demonstrated that the surgical approach exhibited positive effects in terms of improving quality of life and reducing emotional/behavioral problems and school absenteeism in children with PFAPA [77].

IL-1 Antagonists

The rationale behind IL-1 inhibition as a therapeutic approach is the overexpression of inflammasome-related genes and IL-1beta dysregulation during febrile episodes [71-72]. The administration of 1 mg/kg of anakinra (a recombinant IL-1 antagonist) in five patients less than 18 years old on the second day of fever; demonstrated decreased inflammatory markers and clinical improvement [78,79].

Cyclic Neutropenia Treatment

Treatment with a continuous schedule of granulocyte colony-stimulating factor (G-CSF) (filgastrim or pegfilgrastim) is recommended instead of observation alone [80]. The goal of treatment is to maintain the absolute neutrophil count nadir at 500/microL or more, in order to prevent infectious complications. The administration of G-CSF should be done daily or three times a week at an initial dose of 2-3 mcg/kg/day [80]. Supportive care including dental care, maintaining regular schedule immunizations, and bone density monitoring is crucial in reducing the risks of infection and preventing side effects of G-CSF treatment.

Monogenic Periodic Fever syndromes (CAPS, Familial Mediterranean Fever, MKD/HIDS, and TRAPS) Treatment 

CAPS: The use of three IL-1 antagonists is currently approved for the management of CAPS [2]: anakinra from the age of 8 months, rilonacept from the age of two years, and canakinumab from the age of two years

Familial Mediterranean Fever: The cornerstone of treatment in familial Mediterranean fever is the lifelong administration of colchicine. It has been documented that the risk of amyloidosis is considerably reduced [2]. The initial dose proposed is the following [81]: 0.5 mg/day for children less than five years of age, 0.5 to 1 mg/day for children 5-10 years old, and 1-1.5 mg/day for children more than 10 years old.

MKD/HIDS: Treatment of this condition is challenging. Patients might alleviate attacks with non-steroidal anti-inflammatory drugs (NSAIDs) combined with corticosteroids. Colchicine seems to be ineffective, hence is not recommended [78]. For patients that experience severe and recurrent attacks maintenance therapy with IL-1 blockade or etanercept is recommended. IL-6 blockade with tocilizumab might be effective in patients that are refractory to other treatments. Hematopoietic cell transplant is used as a last resort for patients that did not respond to previously mentioned therapies [2,78].

TRAPS: Most of the therapeutic recommendations are based on cohort studies and several randomized controlled trials [2]. NSAIDS provide symptomatic relief in 75% of patients. However, for attack termination, corticosteroids are needed at a 0.5-1 mg/kg dose and the effect may disappear with time. The role of IL-1 antagonists in the treatment of frequent attacks has been well documented. The Canakinumab Pivotal Umbrella Study in Three Hereditary Periodic Fevers (CLUSTER) study described the effectiveness of canakinumab at a dose of 2mg/kg every four weeks [82]. Anakinra is another cost-effective alternative; however, its half-life is only four hours.

## Conclusions

We emphasize the importance of considering the age at onset, family history, duration of febrile episodes, length of interval between episodes, associated symptoms, and treatment response when investigating periodic fever. Many infectious causes of periodic fever are generally infrequent in Western nations, with the exception of repeated separate uncomplicated infections; consequently, doctors should be aware of suggestive case history data. Immune-mediated and autoinflammatory illnesses should be considered once an infectious or neoplastic cause has been ruled out. If steroid medication is being considered, it is critical to rule out the potential of an infectious disease or a tumor. A detailed physical exam during and between febrile episodes, in combination with case history data, may provide important hints and direct laboratory tests. A persistent periodic fever, however, may remain idiopathic after a comprehensive examination. Since new indications and symptoms may arise over time, a careful follow-up is required in these children. Due to the physiological vulnerability to infections that is typical of the pediatric age group, most children with recurrent fever will have self-limited, common diseases and will have a fair prognosis. We recommend careful diagnostic protocols to be set in place to rule out possible life-threatening causes, and more randomized control trials (RCTs) to improve treatment modalities for periodic fever of unknown origin in the pediatric population.
